# A tumor-targeting cRGD-EGFR siRNA conjugate and its anti-tumor effect on glioblastoma *in vitro* and *in vivo*

**DOI:** 10.1080/10717544.2016.1267821

**Published:** 2017-02-09

**Authors:** Shuai He, Bohong Cen, Lumin Liao, Zhen Wang, Yixin Qin, Zhuomin Wu, Wenjie Liao, Zhongyi Zhang, Aimin Ji

**Affiliations:** 1 Department of Pharmacy, Zhujiang Hospital of Southern Medical University, Guangzhou, China and; 2 Guangdong Provincial Key Laboratory of New Drug Screening, School of Pharmaceutical Sciences, Southern Medical University, Guangzhou, China

**Keywords:** siRNA delivery, conjugate system, tumor targeting, gene silencing, glioblastoma

## Abstract

The epidermal growth factor receptor (EGFR) is an important anti-tumor target. The development of novel molecular-targeted anti-tumor drugs that can target the interior of tumor cells and specifically silence EGFR expression is valuable and promising. In this work, a promising anti-tumor conjugate comprising methoxy-modified EGFR siRNA and cyclic arginine-glycine-aspartic acid (cRGD) peptides, which selectively bind to αvβ3 integrins, was synthesized and examined. To prepare cRGD-EGFR siRNA (cRGD-siEGFR), cRGD was covalently conjugated to the 5′-end of an siRNA sense strand using a thiol-maleimide linker. The cellular uptake and cytotoxicity of cRGD-siEGFR *in vitro* were tested using an αvβ3-positive U87MG cell line. *In vivo* bio-distribution, anti-tumor activity, immunogenicity and toxicity were investigated in a nude mouse tumor model through repeated i.v. administration of cRGD-siEGFR (7 times over a 48 h interval). Analyses of *in vitro* data showed that cRGD-siEGFR silenced EGFR expression effectively, with high tumor targeting ability. Administration of cRGD-siEGFR to tumor-bearing nude mice led to significant inhibition of tumor growth, obvious reduction of EGFR expression and down-regulation of EGFR mRNA and protein in tumor tissue. Furthermore, serum biochemistry and pathological section evaluation did not indicate any serious toxicity of cRGD-siEGFR *in vivo*. cRGD-siEGFR is likely a promising candidate with high targeting ability, substantial anti-tumor effects and low toxicity *in vitro* and *in vivo*.

## Introduction

Glioblastoma (GBM; WHO grade IV astrocytoma) is the most common and aggressive primary malignant tumor of the CNS (Louis, 2006). Current conventional treatments for glioblastoma include surgery, radiotherapy and chemotherapy, while treatment effects are limited, with adverse reactions and injury to the human body. Recently, novel molecular-targeting drugs have been applied as a primary approach to glioblastoma treatment and exhibit high specificity, lower toxicity and fewer side effects and good tolerance (Le Tourneau et al., 2015; Cetin et al., 2016).

Epidermal growth factor receptor (EGFR) has been shown to be overexpressed in a variety of tumors and is one of the significant factors responsible for the development of gliomas. The EGFR tyrosine kinase inhibitors (EGFR-TKIs) have become an important focus for tumor-related drug development (Xu et al., 2016), such as small-molecule targeted drugs (Maemondo et al., 2010; Horiike et al., 2014; Liang et al., 2014; Choi et al., 2015), monoclonal antibodies (Hudis, 2007; Jonker et al., 2014; Pietrantonio et al. 2013) and cancer vaccines (Guo et al., 2013; Ahmed & Bae, 2014). However, small-molecule targeted inhibitors and monoclonal antibody drugs cause several adverse reactions, primarily because EGFR expression is suppressed in normal cells in addition to tumor cells. Drug resistance is primarily related to EGFR-associated proteins that are prone to isomerization, thereby reducing the binding of the drug. Therefore, the development of novel tumor-targeting drugs for EGFR has become more and more urgent.

Previous studies have shown that the αvβ3 integrin receptor is highly expressed on the cell surface in multiple malignant tumors and tumor blood vessels, while barely expressed in normal cells and tissues (Shimaoka et al., 2003; Wang et al., 2016). Small interfering RNA (siRNA) has great therapeutic potential for cancers caused by abnormal gene overexpression or mutations *via* sequence-specific post-transcriptional gene silencing (Shen et al., 2014). However, to activate the RNAi pathway, siRNA molecules require safe and efficient delivery systems, such as nanoparticles and conjugates (Shen et al., 2013; Liu et al., 2014), which enable prolonged circulation *in vivo*, high accessibility to target cells and optimized cytosolic release after efficient cellular uptake (Li et al., 2016). In our previous studies (Liu et al., 2014), cRGD-Vegfr2 siRNA conjugates were synthesized. *In vitro* and *in vivo* studies showed that cRGD-Vegfr2 siRNA could silence the expression of Vegfr2 mRNA and inhibit tumor angiogenesis.

However, little effort has been spent on the development of suppressing EGFR expression with siRNA conjugates for glioblastoma therapy. Here, cRGD-siEGFR conjugates have been synthesized, based on the high affinity of integrin αvβ3 to cRGD (Dechantsreiter et al., 1999). A cRGD peptide was covalently attached to the end of a sense strand of siRNA, which silences EGFR mRNA. The anti-tumor effect of cRGD-siEGFR was observed *in vitro* and *in vivo*, including the specific silencing effect, tumor targeting ability, anti-tumor growth activity, toxicity and immune stimulation reaction. Moreover, the feasibility and shortcomings of cRGD-siEGFR for use as a novel molecular-targeted anti-tumor drug were systematically investigated.

Compared with the current small-molecule targeted inhibitors and monoclonal antibody drugs, cRGD-siEGFR may have some beneficial characteristics, such as better tumor targeting and fewer side effects. In addition, cRGD-siEGFR could elicit less drug resistance by inhibiting EGFR expression at the gene level. It could be a valuable and promising way to develop novel molecular-targeted anti-tumor drugs that specifically silence EGFR expression (Lee et al., 2015).

## Materials and methods

### Materials

U87MG (human malignant glioblastoma multiforme cell line, ATCC® number: HTB-14). HeLa cells were kindly provided by Department of Hematology, Zhujiang Hospital, Southern Medical University (Guangzhou, China). Cells were grown and cultured using supplier recommended reagents and media according to the standard protocols and procedures.

SYBR® Premix Ex Taq™ and PrimeScript™ RT reagent Kit with gDNA Eraser (TaKaRa, Kusatsu city, Japanese), 100 bp DNA Ladder (Genscript, Piscataway, NJ, Cat.No.M102R), Trizol reagent and Lipofectamine 2000 (Invitrogen, Carlsbad, CA). Novex® ECL Chemiluminescent Substrate Reagent (Invitrogen). Monoclonal anti-mouse EGFR/Flk-1 antibody (R&D, Shanghai, China), Primary monoclonal anti-integrin αvβ3 antibody (eBioscience, San Diego, CA, Catalog Number: 11-0519), D-Luciferin (BioVision, Milpitas, CA)/ELISA kit (eBioscience).

### siRNA sequence backbone modifications and verification

siRNA sequences for experiments:

Human EGFR siRNA (Sense strand: 5′-CAAAGUGUGUAACGGAAUAdTdT-3′; Anti-sense strand: 5′-UAUUCCGUUACACACUUUGdTdT-3′)

Negative control siRNA (Sense strand: 5′-AUCGAAUUCCUGCAGCCCGUUdTdT-3′; Anti-sense strand: 5′-AACGGGCUGCAGGAAUUCGAUdTdT-3′)

Mouse Vegfr2 siRNA (Sense strand: 5′-CGGAGAAGAAUGUGGUUAAdTdT-3′; Anti-sense strand: 5′-UUAACCACAUUCUUCUCCGdTdT-3′)

Primer sequences for qRT-PCR analysis:

Human EGFR (Forward primer: 5′-GCCGCAAAGTGTGTAACGGAATAG-3′; Reverse primer: 5′-TGGATCCAGAGGAGGAGTATGTGT-3′)

Human GAPDH(Forward primer: 5′-CGGAGTCAACGGATTTGGTCGTAT-3′; Reverse primer: 5′-AGCCTTCTCCATGGTGGTGAAGAC-3′)

Indodicarbocyanine-5 (Cy5)-labeled siRNA (siRNA-Cy5) and all of the abovementioned siRNAs were purchased from Guangzhou RiboBio Co., Ltd.

The relative expression level of EGFR mRNA was tested by qRT-PCR. In order to improve the stability, reduce the immunogenicity and off-target effect, EGFR siRNA sequence was modified as seen in Table S1.

### Synthesis of cRGD-siEGFR

To prepare cRGD-siRNA molecules, cyclic RGD was covalently conjugated to the 5′-end of an siRNA sense strand using a thiol-maleimide linker. The synthetic process was performed as previously described (Liu et al., 2014). The molecular weight of cRGD-sense strand siRNA was characterized by Oligo HTCS LC–MS system (Novatia, Newtown, Pennsylvania). The purity of conjugated cRGD-siRNA was determined by HPLC. The analyses were performed employing an Agela C-8 column (25 cm × 4.6 mm) according to the following conditions: starting from 0.1 M triethylammonium acetate pH 7.4, a linear gradient of 0%–50% MeCN was pumped at a flow rate of 1 mL/min for 0 min–30 min.

### Serum stability of cRGD-siEGFR and EGFR siRNA

To improve the stability, the EGFR siRNA sequence was modified, as seen in Table S1. Five microliters of 20 μM cRGD-siEGFR or EGFR siRNA was mixed with 5 μL of mouse serum and incubated at 37 °C for 0, 12, 24, 36 and 48 h. Aliquots were taken at each of the time points and subjected to electrophoresis in 1.2% non-denaturing agarose gels.

### qRT-PCR and western blot analysis

The qRT-PCR and western blot analysis were conducted as previously described (Liu et al., 2014).

### Cytotoxicity analysis *in vitro*

Cells were seeded into 96-well microtiter plates (BD Falcon) at a density of 4 × 10^4^ cells/well and allowed to attach for 24 h. cRGD-Nonsense Control siRNA (cRGD-siNC) at concentrations of 100, 200, 500, 1000, 1500 and 2000 nM (final concentration) were added into different wells in triplicate and incubated with cells for 24 h, 48 h and 72 h, according to the manufacturer’s protocols. Then, 10 μL of Cell Counting Kit-8 (Dojindo, Japan) solution was added to each well. Plates were incubated at 37 °C for an additional 1 h, and optical densities were recorded at 450 nm using a microplate reader (Bio-Rad, Hercules, CA). Cell viability was plotted as a percentage of untreated control cells.

### The expression level of integrin αvβ3

U87MG cells were transfected with Cy5-labeled cRGD-siEGFR, Cy5-labeled siRNA and Lipo2000/siRNA-Cy5 complexes in Dulbecco’s modified Eagle’s medium (DMEM) containing 10% fetal bovine serum (FBS). To demonstrate the effect of cRGD binding to the αvβ3 receptor, the U87MG cells were also pre-treated with 1 μM of un-conjugated cRGD peptide (cRGD blocked) for 30 min at 37 °C prior to transfection with cRGD-siEGFR-Cy5, as shown in a previous report (Alam et al., 2011). The final concentration of the compound was 100 nM. Six hours after transfection, cells were washed with PBS and fixed immediately using 4% paraformaldehyde at room temperature for 15 min, followed by nuclear staining with 4,6-diamidino-2-phenylindole (DAPI) (Roche, Switzerland) for 10 min at 37 °C. Cells were imaged with confocal microscopy (Olympus, Tokyo, Japan; Cy5 excitation = 640 nm, emission = 680 nm).

### Cellular uptake level

U87MG cells were incubated with different concentrations of cRGD-siEGFR-Cy5 (100 nM or 400 nM) and Lipo2000/siRNA-Cy5 complexes (100 nM) for 6 h at 37 °C. Then, the cells were washed with PBS three times to remove any extracellular cRGD-siEGFR-Cy5 or siRNA-Cy5. Cells were collected and analyzed with a BD FACS Calibur Cell Sorting System (BD Biosciences, San Jose, CA). Data were obtained and analyzed using Cell Quest software.

### Apoptosis analysis

Briefly, 72 h after siRNA transfection, U87MG cells were stained in accordance with the manufacturer’s instructions. Adherent and floating cells were collected, stained with FITC-labeled Annexin V (eBioscience) and propidium iodide (eBioscience), and analyzed on a BD FACS Calibur Cell Sorting System (BD Biosciences).

### Cell proliferation analysis by CCK-8 and 5-ethynyl-2′-deoxyuridine (EdU) assays

Cells were seeded into 96-well microtiter plates (BD Falcon, San Jose, CA) at a density of 4 × 10^4^ cells/well and allowed to attach for 24 h. cRGD-siEGFR at concentrations of 0, 400, 600 or 800 nM (final concentration) were added into different wells in triplicate and incubated with cells for 48 h and 72 h, according to the manufacturer’s protocols. Then, 10 μL of CCK-8 (Dojindo, Japan) solution was added to each well. Plates were incubated at 37 °C for an additional 1 h and optical densities were recorded at 450 nm using a microplate reader (Bio-Rad). Cell viability was plotted as a percentage of untreated control cells.cRGD-siEGFR, at a concentration of 800 nM, was added and incubated with cells for 48 h. Then, 50 μM EdU (Guangzhou Ruibo, China) solution was added to each well and incubated at 37 °C for an additional 2 h. EdU labeling and EdU staining were performed using a standard protocol (Salic & Mitchison, 2008). EdU-stained cells were counterstained with Hoechst and imaged with confocal laser scanning microscopy.

### Tumor model establishment

BALB/c nude mice (female, 4–6 weeks old, ∼20 g) were purchased from the Experimental Animal Center of Sun Yat-Sen University and maintained in a sterile environment, according to the standardized animal care guidelines. The experiments were performed according to the national regulations. Nude mice were inoculated subcutaneously on the right back with 5 × 10^6^ U87MG cells. When tumor volume reached 150 mm^3^, the animals were randomized into different groups for anti-tumor activity. For bio-distribution and tumor vascular permeability experiments with cRGD-siRNA, 5 × 10^6^ U87MG or HeLa cells were injected subcutaneously on the right back of nude mice, and experiments were performed when tumor volume reached 120 mm^3^.

### *In vivo* distribution

Mice bearing U87MG tumors were injected intravenously with 1 nmol/20 g cRGD-siRNA-Cy5 or siRNA-Cy5 at single doses (*n* = 4). The subsequent bio-distribution was detected at 12 h, 24 h, 48 h and 72 h using an IVIS Spectrum imaging system at the appropriate wavelength (Cy5: λex = 640 nm, λem = 680 nm). The tumors and major organs were excised and imaged at 24 h or 72 h.

### Tumor vascular permeability

Mice bearing U87MG tumors were injected intravenously with cRGD-siRNA-Cy5 or siRNA-Cy5. Mice bearing HeLa tumors were injected with cRGD-siRNA-Cy5. Animals were euthanized 24 h after treatment. Immunofluorescence analysis was performed as previously described (Liu et al., 2014).

### Anti-tumor activity

Mice bearing U87MG tumors were injected intravenously with cRGD-siEGFR 7 times over a 48 h interval with one of the following treatments. A: saline, B: cRGD-siNC (5 nmol/20 g), C: cRGD-Vegfr2 siRNA (1.5 nmol/20 g), D: cRGD-siEGFR (1.5 nmol/20 g), E: cRGD-Vegfr2 siRNA (1.5 nmol/20 g) + cRGD-siEGFR (1.5 nmol/20 g), F: cRGD-siEGFR (5 nmol/20 g). Tumor volumes were measured with a caliper before injection and calculated using the following formula: volume = ½ × length × (width)^2^, where length represented the longest tumor diameter and width represented the shortest tumor diameter. The growth curves were plotted as the mean tumor volume ± SD (standard deviation). Animals were euthanized 3 days after the last treatment and the tumors and visceral organs were excised and preserved in liquid nitrogen for further analysis.

EGFR expression level was determined by qRT-PCR, western blot and immunohistochemistry. Tissue sections were processed for TUNEL analysis using an *in situ* cell death detection kit-POD (Roche) as a measure of apoptosis.

### Toxicity and immunogenicity evaluation *in vivo*

For immune response studies, the serum was collected from C57BL/6J mice 6 h after injection of cRGD-siEGFR (5 nmol/20 g) or saline. IL-6, IL-12, IFN-α and IFN-γ levels in the serum were detected by ELISA. For toxicity studies, the serum was collected from mice bearing U87MG tumors that were injected intravenously with cRGD-siEGFR 7 times, and ALT (alanine aminotransferase) and Cr (creatinine) were measured with an automated Aeroset Chemistry Analyzer (Abbott, Abbott Park, IL), based on manufacturer’s instructions.

### Visceral organ toxicity check

For the visceral organ toxicity analysis, 3 days after the last of seven injections, the heart, liver, spleen and kidneys were excised from nude mice treated with cRGD-siEGFR (1.5 nmol/20 g), cRGD-siEGFR (5 nmol/20 g) or saline. Tumor tissues were fixed immediately using 4% paraformaldehyde. All the sections were stained with hematoxylin eosin (HE).

### Statistical analysis

Statistical analysis of the data was performed through one-way analysis of variance (ANOVA) (SPSS software, version 19.0). The results are expressed as the mean ± standard error, and *p *<* *0.05 was considered statistically significant. All statistical tests were two-sided.

## Results

### Synthesis and serum stability of cRGD-siEGFR

**Figure 1.**
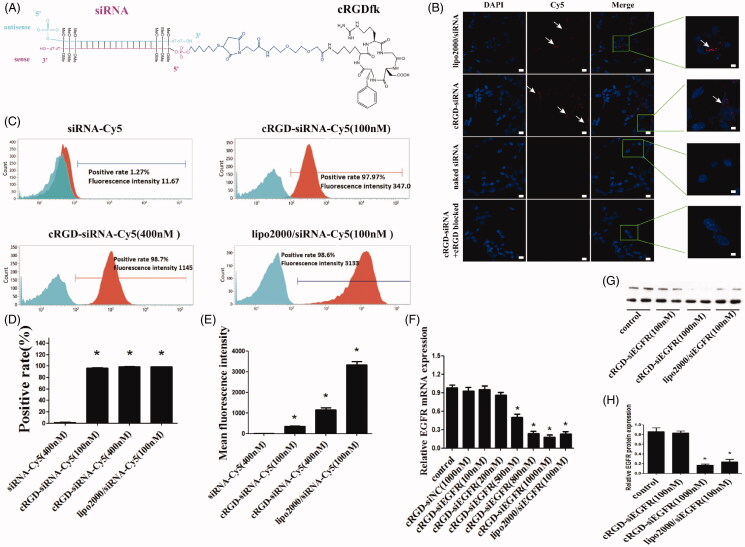
The schematic depiction, cell distribution, uptake and gene silencing efficiency of cRGD-siEGFR *in vitro*. (A) A diagram of cRGD-siEGFR conjugates. The cRGD moiety is conjugated to the 5′-phosphate of the passenger (sense) strand of EGFR siRNA through a thiol-maleimide linker. The backbone of EGFR-siRNA was modified with three 2′-O-Me on both ends of the sense strand and antisense strand. (B) Confocal laser scanning microscopy images of the intracellular distribution of cRGD-siEGFR. U87MG cells were transfected with Lipo2000/siRNA-Cy5, cRGD-siEGFR-Cy5, naked EGFR siRNA-Cy5, and for additional specificity tests, before being transfected with cRGD-siEGFR-Cy5, cells were pre-treated with 1 μM un-conjugated cRGD peptide (cRGD blocked). Cell nuclei were counterstained with DAPI (blue) and siRNA was labeled with Cy5 (red; marked by arrow). After 6 h of transfection, cells were fixed and visualized using confocal laser microscopy. (C) Cellular uptake levels of cRGD-siEGFR-Cy5 and siRNA-Cy5 in U87MG cells after 6 h of incubation with different ratios of cRGD-siEGFR-Cy5 and siRNA-Cy5, as measured by flow cytometry. Red area: fluorescence intensity related to cellular uptake of siRNA-Cy5. (D and E) Quantitative analysis of Cy5-positive expression levels and fluorescence intensity, *p < 0.05, compared with the siRNA-Cy5 group, *n* = 3. (F) Specific gene silencing *in vitro* for cRGD-siEGFR. U87MG cells were treated for 48 h with different concentrations of cRGD-siEGFR. (G and H) Quantitative analysis of EGFR protein expression levels. The expression of EGFR protein was calculated relative to the expression of GAPDH protein. *p <* *0.05 vs. control group, #*p *<* *0.05 vs. cRGD-siNC group, *n* = 3; bar = 20 μm or 5 μm.

A diagram of cRGD-siRNA conjugates is shown in Figure 1(A). The cRGD moiety was conjugated to the 5′-phosphate of a passenger strand of siRNA through a thiol-maleimide linker. HPLC-MS results showed that the molecular mass of synthesized cRGD-siEGFR was consistent with the theoretical molecular mass. The cRGD-conjugated sense strand siRNA was determined to be 7923.4 Da, which was acceptably close to the predicted mass of 7921.4 Da, as seen in Supplementary Figure S1(A). The RP-HPLC results showed that the purity of cRGD-siEGFR reached 88.0%, as seen in Supplementary Figure S1(B). For EGFR siRNA sequence and backbone modification verification, the silencing efficiency of EGFR siRNA-C was measured and determined to be over 80%, and the stability was obviously improved, without decreasing the silencing efficiency, by the use of three 2′-O-Me modifications on the both ends of the sense strand and antisense strand of siRNAs, as seen in Figure S1(C,D) and Table S1. Incubation of 2′-O-Me-modified cRGD-siRNA conjugates in mouse serum revealed very little degradation after 48 h. In similar conditions, unmodified RNA oligonucleotides were mostly degraded after 24 h, as seen in Figure S1(C).

### Intracellular distribution of cRGD-siEGFR

To detect the intracellular distribution, cRGD-siEGFR conjugates were labeled with a fluorophore (Cy5). cRGD-siEGFR delivered siRNA into U87MG cells, while naked siRNA did not. When integrin αvβ3 receptor on the cell surface was blocked by pre-incubation with cRGD, cRGD-siEGFR failed to enter cells, which suggested that cRGD-siEGFR was specifically taken up by glioblastoma cells *via* αvβ3 receptors (Figure 1B). The integrin αvβ3 expression level of U87 MG cells was 99.98% (data not shown).

### Cellular uptake of cRGD-siEGFR

Flow cytometry results (Figure 1C–E) showed that naked siRNA barely entered U87MG cells, with a Cy5-positive rate of 1.27% and a fluorescence intensity of 11.67, in accordance with the results of confocal microscopy. Compared with the naked siRNA-Cy5 group, U87MG cells had better ability to take up cRGD-siEGFR-Cy5 (100 nM), cRGD-siEGFR-Cy5 (400 nM) and Lipo2000/siRNA-Cy5 (100 nM), and the positive rate of uptake was 97.97%, 98.68% and 98.58%, respectively, and the fluorescence intensity was 347, 1145 and 3133, respectively. The uptake ability increased as the administration dose increased.

### Gene knockdown efficiency of cRGD-siEGFR *in vitro*

Compared with the control group, EGFR mRNA in groups transfected with 500 nM, 800 nM, and 1000 nM cRGD-siEGFR decreased significantly (the expression level was 47.39%, 23.93%, 18.34%, respectively, *p *<* *0.01, Figure 1F). In terms of the amount of conjugate used for transfections, the silencing efficacy of cRGD-siEGFR increased as the concentration rose; when the concentration reached 1000 nM, the silencing efficacy was higher than the Lipo2000/siRNA group, and the difference was significant (*p *<* *0.05). Total protein was collected for western blot analysis after 72 h, and EGFR protein expression was significantly decreased in the cRGD-siEGFR group and Lipo2000/siRNA group, compared with control groups and the cRGD-siNC group (Figure 1G,H). Taken together, these results suggested that cRGD-siEGFR effectively inhibited EGFR expression in U87MG cells.

### Inhibition of cell proliferation and promotion of apoptosis by cRGD-siEGFR *in vitro*

**Figure 2.**
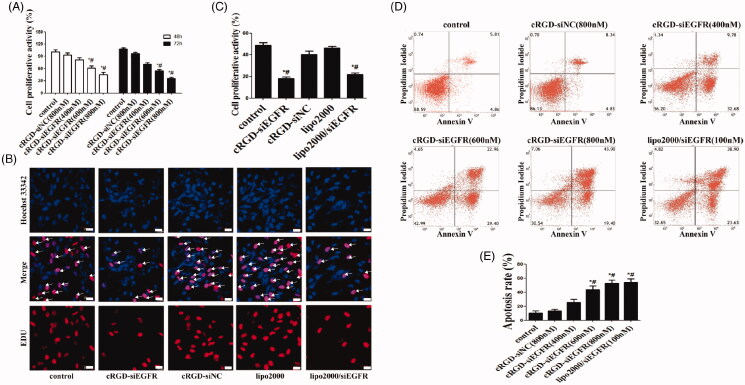
Cell proliferation and apoptosis *in vitro*. (A) Cell proliferation was detected with CCK-8. U87MG cells were incubated with different dosages of cRGD-siEGFR (400, 600 or 800 nM) and tested at 48 h or 72 h by a CCK-8 assay. Control was the untreated group. cRGD-siNC (800 nM) was the negative control group. (B) The proliferation of U87MG cells was detected by EDU after treatment with cRGD-siEGFR (800 nM) for 48 h. Control was the untreated group. cRGD-siNC (800 nM) was the negative control group. Treatment with siRNA (100 nM) using a transfection reagent (Lipo2000) was used as a knockdown positive control. A fluorescence image of cellular DNA (Hoechst stain; blue), and a fluorescence image of EdU-labeled DNA revealed by reaction with Alexa568 azide (red; marked by arrow). (C) Quantitative analysis of U87MG cell proliferation. The proliferative activity was relative to the fluorescence image of EdU-labeled DNA (red fluorescence; marked by arrow). (D) The apoptosis of U87MG cells was detected by flow cytometry after different treatments for 3 days. (E) Quantitative analysis of U87MG cell apoptosis. *p <* *0.05 vs. Control group, #*p* < 0.05 vs. cRGD-siNC group, *n* = 3; bar = 20 μm.

CCK-8 assay results showed that cRGD-siEGFR at different concentrations (600 nM and 800 nM) significantly inhibited the proliferation of U87MG cells at 48 h and 72 h (*p *<* *0.01, Figure 2A). The inhibition of proliferation *in vitro* was also confirmed with an EDU experiment (Figure 2B,C).

Flow cytometry results showed that cRGD-siEGFR at different concentrations (400 nM, 600 nM and 800 nM) clearly induced apoptosis of U87MG cells *in vitro*, with apoptosis rates of 26.59%, 45.23% and 55.28%, respectively (Figure 2D,E). However, the results of the CCK-8 experiment showed that cRGD-siNC was of very low toxicity, as seen in supplementary Figure S2.

### Bio-distribution and tumor vascular permeability of cRGD-siEGFR *in vivo*

**Figure 3.**
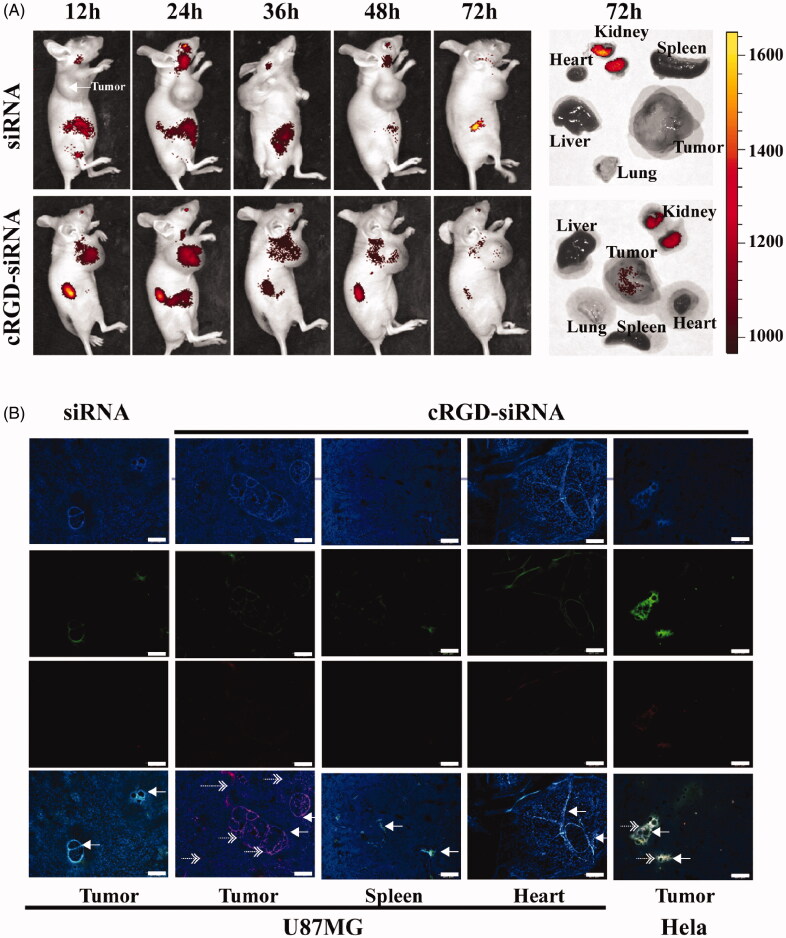
*In vivo* distribution and tumor vascular permeability of cRGD-siEGFR. (A) cRGD-siEGFR and un-conjugated siRNA bio-distributes to tumors. Nude mice bearing a U87MG tumor xenograft were injected with Cy5-labeled cRGD-siRNA conjugates or un-conjugated 2′-O-Me-stabilized siRNAs (tail vein, single dose, 1 nmol/20 g), and fluorescence images of whole animals or isolated organs were taken at indicated time points, 72 h after injection, using an IVIS imaging system. All images were scaled to the same minimum and maximum color values. (B) Tumor tissue targeting of cRGD-siRNA. Nude mice (female, 4–6 weeks, ∼20 g) were inoculated subcutaneously on the right back with 5 × 10^6^ U87MG or HeLa cells. When tumor volume reached 120 mm^3^, the animals were randomized into different groups for treatment testing. Mice bearing U87MG tumors were injected with either cRGD-siEGFR-Cy5 (1 nmol/20 g) or EGFR siRNA-Cy5 (1 nmol/20 g). Mice bearing HeLa tumors were injected with cRGD-siEGFR-Cy5 (1 nmol/20 g). Animals were euthanized 24 h after treatment. Tumor tissue was stained with DAPI (blue-fluorescence), blood vessels were marked with CD31 (green-fluorescence; marked by left arrow), and siRNA was labeled with Cy5 (red fluorescence; marked by right arrow); bar = 200 μm.

The results of *in vivo* imaging of tumor-bearing mice showed that cRGD-siEGFR-Cy5 could specifically target tumors after intravenous injection (1 nmol/20 g). At 12 h and 24 h, a large amount of Cy5 fluorescence was observed at the tumor site; fluorescence was also observed in kidney tissue, as well as a small amount in liver tissue. However, 12 h to 72 h after injection with siRNA-Cy5, mice exhibited no Cy5 fluorescence at the tumor location. After dissection, consistent results were found, as shown in the images of organs and tissues *in vivo*. Both of the results indicated that cRGD-siEGFR could specifically target tumor tissue (Figure 3A).

After intravenous injection, cRGD-siEGFR-Cy5 could permeate into tumor stroma, while siRNA-Cy5 failed to enter tumor stroma (Figure 3B). In normal tissue, without expression of ανβ3 receptors, and HeLa tumor tissue, cRGD-siEGFR failed to reach the tumor stroma. The integrin αvβ3 expression level of HeLa cells was 4.29% (data not shown).

### Anti-tumor activity of cRGD-siEGFR

**Figure 4.**
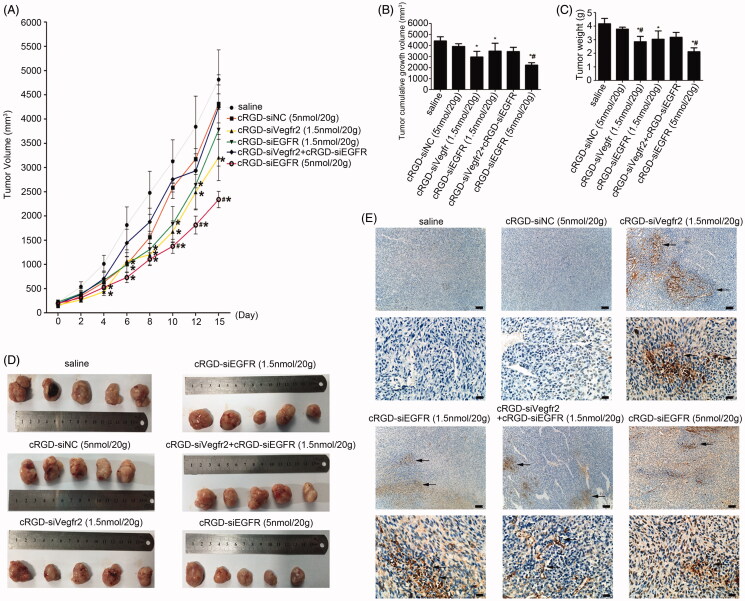
Anti-tumor activity of cRGD-siEGFR *in vivo*. (A) The tumor growth curve. Nude mice were repeatedly administered treatments (7 times) *via* intravenous injection in the tail, over an interval of 2 days. The tumor volume was measured before each injection. The growth curves were plotted as the mean tumor volume ± SD (standard deviation). (B) The cumulative growth of tumor volume. The cumulative growth volume is equal to the volume of a tumor three days after the last injection minus the volume of the tumor before injection. (C) Tumor weight. The mice were euthanized three days after the last injection, and the tumor was excised, weighed and photographed (D). (E) Apoptosis in tumor tissue was detected by TUNEL staining. The apoptotic cells were brown under a light microscope; bar = 100 μm or 20 μm.

The tumor-bearing mice were administered treatments 7 times *via* intravenous injection in the tail over a period of 48 h. The tumor volume and body weight were measured before injection. On the third day after the last administration, the tumor volume and weight were measured again. The tumor growth curve is shown in Figure 4(A). There was no significant difference between the saline and cRGD-siNC treated groups, which demonstrated that cRGD-siNC did not have specific anti-tumor effects. Compared with saline-treated groups, cRGD-siEGFR (5 nmol/20 g) and cRGD-Vegfr2 siRNA (1.5 nmol/20 g) inhibited tumor growth significantly, beginning at 4 days after the second injection (*p *<* *0.05), and low-dose cRGD-siEGFR (1.5 nM/20 g) inhibited tumor growth significantly beginning at 6 days after the third injection (*p *<* *0.05).

The mice were euthanized three days after the last injection, and the tumors were excised and weighed (Figure 4B–D). The results showed that high-dose cRGD-siEGFR (5 nmol/20 g), cRGD-Vegfr2 siRNA (1.5 nmol/20 g) and low-dose cRGD-siEGFR (1.5 nmol/20 g) significantly inhibited tumor growth, compared with the saline group; the weight or cumulative growth of the volume of tumors was reduced by approximately 50%, 32% and 27%, respectively. However, the nude mice treated with cRGD-Vegfr2 siRNA (1.5 nmol/20 g) combined with cRGD-siEGFR (1.5 nmol/20 g) had only slight inhibition of tumor growth (*p* > 0.05).

**Figure 5.**
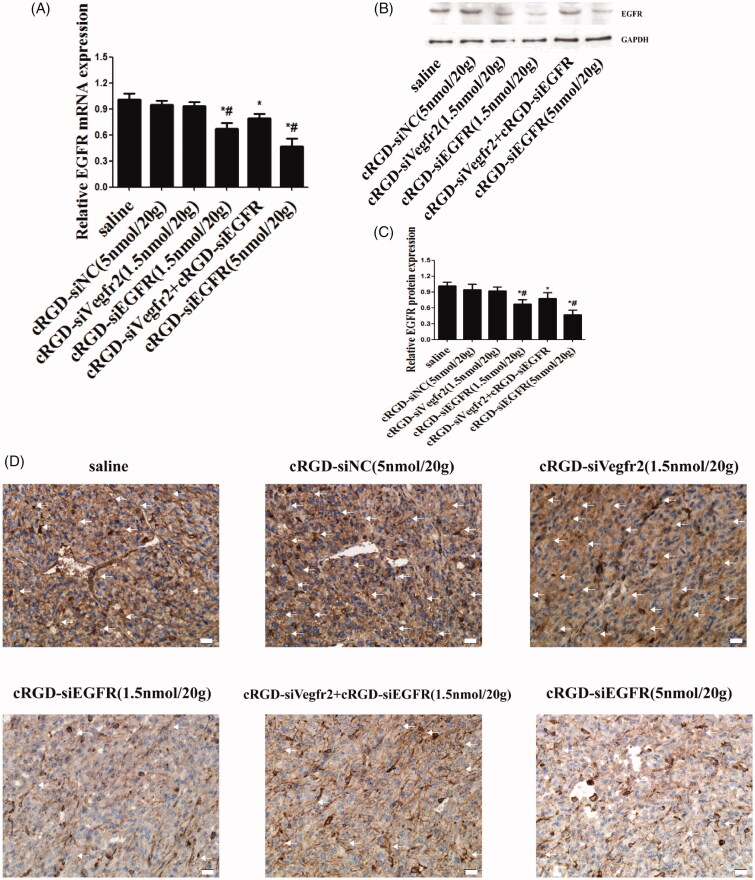
Gene silencing of cRGD-siEGFR *in vivo*. (A) qRT-PCR analysis of the EGFR mRNA levels expressed in tumors. EGFR mRNA expression was normalized to GAPDH mRNA. (B) Western blot analysis of EGFR protein expressed in tumors. (C) Quantitative analysis of EGFR protein expression levels. The expression of EGFR protein was calculated relative to the expression of GAPDH protein. (D) The relative expression level of EGFR protein in tumor tissue was detected by immunohistochemical staining. Anti-EGFR antibody (ab52894) is shown as brown (marked by arrow). *p <* *0.05 vs. control group, #*p *<* *0.05 vs. cRGD-siNC group, *n* = 3; bar = 20 μm.

The results of qRT-PCR demonstrated an obviously low expression of EGFR mRNA in the cRGD-siEGFR (1.5 nmol/20 g) group and cRGD-siEGFR (5 nmol/20 g) group, when compared with the cRGD-siNC (5 nmol/20 g) or saline-treated group (Figure 5A). Furthermore, Figure 5(B,C) shows that the protein level was significantly decreased in the cRGD-siEGFR (1.5 nmol/20 g) group and cRGD-siEGFR (5 nmol/20 g) group. The results of a TUNEL assay demonstrated an obviously higher number of apoptotic cells in tumors from the mice treated with cRGD-siEGFR (5 nmol/20 g), cRGD-Vegfr2 siRNA (1.5 nmol/20 g) combined with cRGD-siEGFR (1.5 nmol/20 g) and cRGD-siEGFR (5 nmol/20 g), in comparison with mice treated with cRGD-siNC (5 nmol/20 g) or saline (Figure 4E). Immunohistochemical analysis using anti-EGFR antibody labeling also displayed a remarkably lower expression level of EGFR in tumors from mice treated with cRGD-siEGFR (5 nmol/20 g), cRGD-Vegfr2 siRNA combined with cRGD-siEGFR (1.5 nmol/20 g) and cRGD-siEGFR (5 nmol/20 g) compared with the mice treated with cRGD-siNC (5 nmol/20 g) or saline (Figure 5D). Taken together, the data show that cRGD-siEGFR can inhibit tumor growth effectively and specifically when administered by intravenous injection in the tail. ELISA and blood biochemical test results showed that no significant difference in IFN-α, IFN-γ, IL-6, IL-12, Cr and ALT were found between the cRGD-siRNA group and control group (Figure S2B-D), suggesting low immunogenicity and toxicity of cRGD-siEGFR. Pathological section results further confirmed the low toxicity of cRGD-siRNA. Even though the mice were administered 7 times with a high dose (5 nmol/20 g), no serious toxicity to organs was found (Figure S2E).

## Discussion

In this study, the purity of cRGD-siEGFR reached 78.7%–88.0%, but a purity of at least 90% is required if it is to be used as a drug. The low purity was associated with the small production scale (less than 100 nmol/lot). If the production amount was increased to more than 10 μmol/lot, synthetic purity may be significantly improved and be stable at more than 90% because the original impurities would not increase significantly with amplification of the production volume.

Unconjugated siRNA failed to penetrate the cell membrane and enter U87MG cells. However, cRGD-siRNA conjugates could enter cells through specific binding with αvβ3 receptors. When αvβ3 receptors were blocked, cRGD-siRNA failed to enter U87MG cells, suggesting that cRGD-siEGFR had high tumor-targeting ability compared with traditional tumor-targeting drugs. As part of a passive targeting system, nanoparticles and liposomes fail to target tumor cells efficiently and specifically (Kanasty et al., 2013). In this regard, additional biological materials need to be added for the formation of stable nanoparticles and liposomes, which may result in adverse reactions and toxicity (Knudsen et al., 2015). However, compared with nanoparticles and liposomes, the cRGD-siEGFR conjugates were characterized by a definite molecular structure, molecular components and less toxicity. *In vitro* cytotoxicity was tested by exposing U87MG cells to increasing cRGD-siNC concentrations (50, 100, 200, 500, 1000, 1500 and 2000 nM), and this induced very low toxicity. The average viability of the cells was more than 90% at a concentration of 1000 nM (Figure S2A).

Our results showed that EGFR expression in U87MG cells was specifically silenced by cRGD-siEGFR with an IC_50_ of 500 nM. Moreover, the silencing efficacy reached 80% or above at the concentration of 1000 nM. In our previous study (Liu et al., 2014), an experiment was conducted in HUVEC lines with Vegfr2 mRNA as the target, and the IC_50_ of cRGD-siVegfr2 for Vegfr2 mRNA was lower than 100 nM. The inconsistency may be explained by the following reasons: (1) The target was different. Vegfr2 is an epidermal growth factor receptor for neovascularization, while EGFR is an epidermal growth factor receptor for cells; (2) Characteristics of HUVECs and U87MG cells are different. HUVECs are a type of human umbilical vein endothelial cell and the U87MG cell line belongs to a malignant glioma cell line; (3) The difference in the siRNA sequence may affect the silencing efficiency. For instance, some of the sequences may possess a silencing rate of more than 95%, while others may only have approximately 30% (Angart et al., 2013); (4) The difference in the expression level of target mRNA. Further validation supported that, under the same procedures of RNA extraction and 40 cycles of amplification, the expression level of EGFR mRNA in U87MG cells was 3.2 × 10^5^ times that with VEGFR2 mRNA in the HUVEC cell line (data not shown).

The metabolism research suggested that cRGD-siEGFR was effective at targeting tumors and had a high aggregation at tumor sites after 12 h, 24 h and 48 h. The primary organ for metabolism of the conjugates was the kidney, followed by the liver, and some metabolism was observed in other parts of the body. Confocal microscopy also found that cRGD-siEGFR only aggregated around tumor tissues that had high expression of ανβ3 receptors, while no such aggregation was found in normal tissues or tumor tissues with low ανβ3 receptor expression. Compared with normal nanoparticles and liposomes, cRGD-siEGFR highly and specifically targeted tumors (Dahlman et al., 2014; Fehring et al., 2014).

In the current study, a U87MG cell tumor model was subcutaneously injected into nude mice. U87MG is a type of glioma cell line with its origin in the intracalvarium. Whether cRGD can penetrate into intracalvarial tumor tissues, through the blood brain barrier, was not investigated in our study. Zhang et al. demonstrated that cRGD was able to penetrate into intracalvarial tumor tissues, through the blood brain barrier into intracranial gliomas, and the expression of cRGD in tumor tissues was much higher compared with that in normal brain tissue (Zhang et al., 2011; Wang et al., 2015).

In the present study, we designed a cRGD-Vegfr2 siRNA combined with cRGD-siEGFR treatment group, which could theoretically have a synergistic anti-tumor effect. Our previous studies confirmed that cRGD-siVegfr2 can inhibit angiogenesis of tumors, thereby cutting off the oxygen and nutrient supply for tumor tissue, leading to apoptosis of tumor cells and, ultimately, inhibition of tumor growth (Liu et al., 2014). cRGD-siEGFR directly inhibited tumor cell proliferation and induced tumor cell apoptosis, thereby inhibiting tumor growth. The combination of the two might directly and indirectly inhibit tumor growth. The results of this study, however, showed that there was no significant difference in tumor size in the combined drug group compared with the cRGD-siVegfr2 or cRGD-siEGFR alone groups. Even worse, there was no significant difference between the combined treatment group and the control or cRGD-siNC group. The possible reasons for this might include the following two reasons. (1) The dose of both drugs was insufficient. We gave a combined dose of 1.5 nmol/20 g, but the results of the experiment showed that only a dose as high as 5 nmol/20 g significantly suppressed tumor growth. Therefore, a low dose of the two drugs combined could not induce a synergistic effect. Furthermore, because of competitive binding of cRGD-siEGFR and cRGD-siVegfr2 to the ανβ3 receptor, the combination treatment group needs a much higher dose than that in the single drug groups to produce equivalent effects for inhibition of tumor growth. (2) Because cRGD-siVegfr2 inhibited the angiogenesis of tumors, cRGD-siEGFR could not effectively penetrate tumor tissue *via* vessels, which restricted the synergistic effect.

In this study, we examined the immune response of normal mice treated with 5 nmol/20 g of cRGD-siEGFR and tumor-bearing nude mice after administration (7 times) of cRGD-siEGFR by measuring biochemical indicators of liver and renal toxicity. The blood biochemical test and ELISA results showed that no significant difference in Cr, ALT, IL-6, IL-12, IFN-α and IFN-γ were found between the experimental groups and the control group (Figure S2B-D), suggesting low toxicity of cRGD-siEGFR. Blood biochemical indicators only reflected real-time damage, but there was no evidence of already-present injury or damage in the recovery time; therefore, we observed the pathological sections of the organs in each group of nude mice, and no serious toxicity to organs was found, even in the high-dose (5 nmol/20 g) group (Figure S2E). However, some mild adverse effects were found. For example, pathological sections of kidney indicated that the glomerular filtration ability was disturbed in the high-dose (5 nmol/20 g) group. Previous studies have found that glomerular epithelial cells in healthy humans express α1, α3, α5β1, αvβ3 and αvβ5 integrin receptors (Hamerski & Santoro, 1999). This suggests that cRGD-siEGFR can reach the kidney and be taken up by glomerular epithelial cells. In addition, peptides and low molecular weight proteins, such as cRGD peptides, are filtered by glomeruli. Following this, molecules can be reabsorbed into the renal cortex and the surrounding capillaries in proximal tubules (Briat et al., 2012). Hence, the accumulation of cRGD-siEGFR in the kidney and renal toxicity in the renal cortex may be caused by these factors. Tubular epithelial cells are among the non-immune cells that express TLR1, -2, -3, -4 and -6, suggesting that TLRs might contribute to the activation of immune responses in renal injury (Anders, 2004). Injury of glomerular filtration function with the high-dose (5 nmol/20 g) of conjugate probably occurred because the 21-nucleotide siRNA interacted with TLR3 in the glomerular endothelial cells, thus activating the innate immune response and causing kidney damage, resulting in renal toxicity (Kleinman et al., 2008).

Taken together, our results provide strong evidence for the potential use of cRGD-siRNA to target EGFR in glioma as an anticancer therapeutic. However, several studies will need to be conducted to take this therapeutic to the next stage of drug development. These studies might include (i) increasing the potency of siRNA and limiting the total dose delivered to patients to reduce immunogenicity and toxic effects; (ii) extending the dosing interval, which would increase the time available to repair sub-lethal damage; (iii) optimizing the siRNA backbone to reduce the association of the siRNA molecule with TLR3 in renal tubular endothelial cells and, thus, reduce the nephrotoxicity likely caused by the immunogenicity; and (iv) administering the conjugate with other drugs, such as Gelofusine, to reduce renal reabsorption of cRGD-siRNA, thereby reducing renal toxicity (Briat et al., 2012; Lee et al., 2015).

## Conclusions

We have demonstrated that cRGD-siEGFR can effectively knockdown EGFR expression with high tumor uptake *in vitro* and *in vivo*. In addition, after systemic intravenous delivery of cRGD-siEGFR, we showed that down-regulation of EGFR in mouse tumors could substantially slow tumor growth, and low toxicity or innate immune response was induced *in vivo*. Collectively, cRGD-siEGFR represents a novel tumor-targeting delivery system for siRNAs and a promising candidate for cancer therapy.

## Supplementary Material

Table_with_caption.docx

supplemental_figures.docx

## Notes

**Supplemental data available online**
