# Supplementary material for: A tumor-targeting cRGD-EGFR siRNA conjugate and its anti-tumor effect on glioblastoma in vitro and in vivo
Source: Drug Deliv. 2017 Feb 9;24(1):471–81. doi: 10.1080/10717544.2016.1267821 (PMC8241002; doi:10.1080/10717544.2016.1267821)
Supplement: supplemental_figures.docx [file IDRD_A_1267821_SM8557.docx]

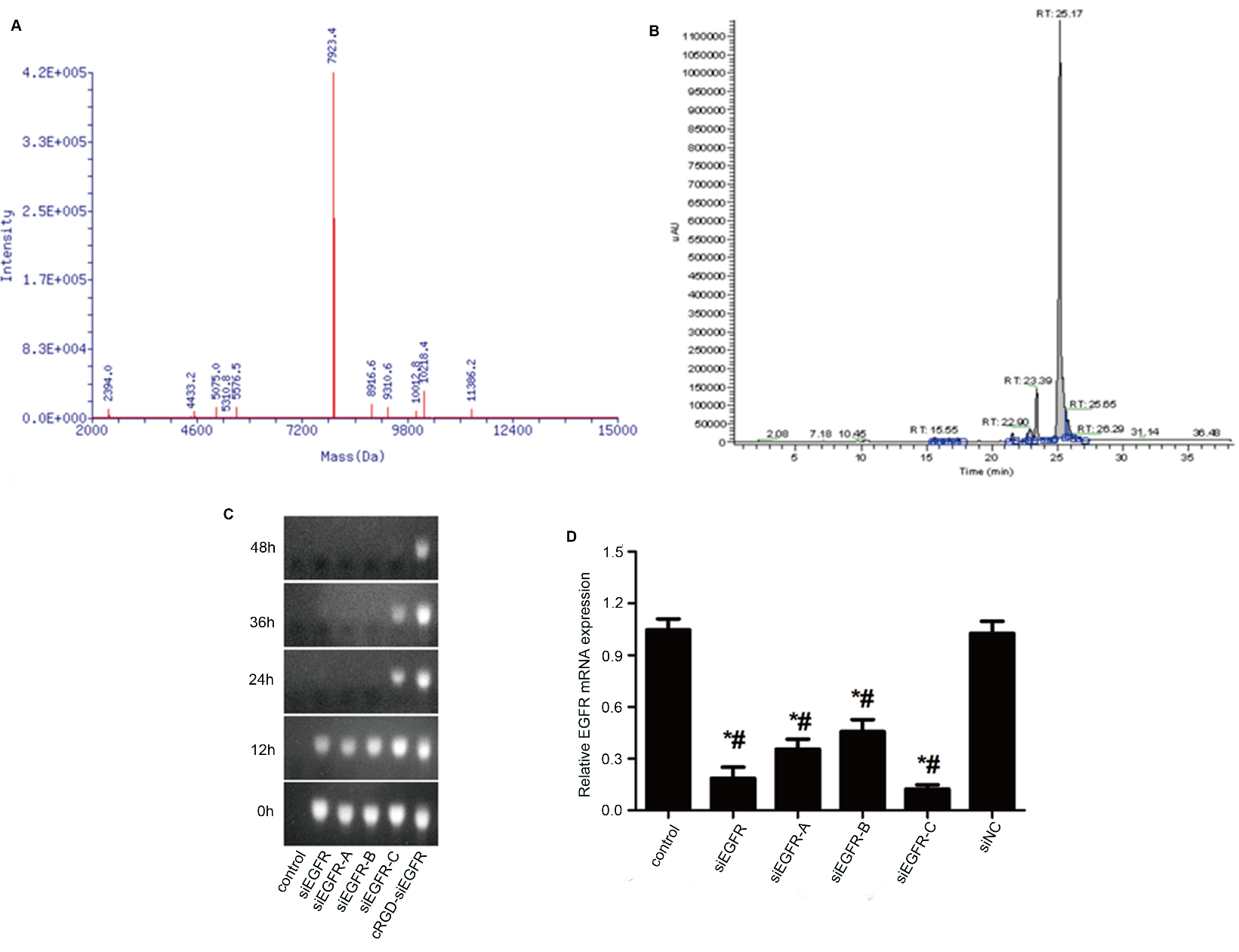


**Figure S1.** The characterization of cRGD-siEGFR conjugates and silencing efficiency of EGFR siRNA sequences. **(A)** The identification of cRGD-siEGFR by HPLC-MS. The measured mass of the sense strand conjugated with cRGD is 7923.4 Da (predicted mass is 7921.4 Da). **(B)** The analysis of purified cRGD-siEGFR with RP-HPLC. The purity of cRGD-siEGFR, calculated by the peak area normalization method, was 88.0%. **(C)** The serum stability of cRGD-siEGFR and EGFR siRNA sequences. Molecules were incubated in mouse serum at 37°C for the indicated time periods and then analyzed using agarose (1.2%) gel electrophoresis. **(D)** Gene silencing efficiency. U87MG cells were treated for 48 h with EGFR-targeting siRNAs, complexed with Lipofectamine 2000. The expression level of EGFR mRNA was analyzed by qRT-PCR. * *P*<0.05 vs. control group, # *P*<0.05 vs. siNC group, n=3


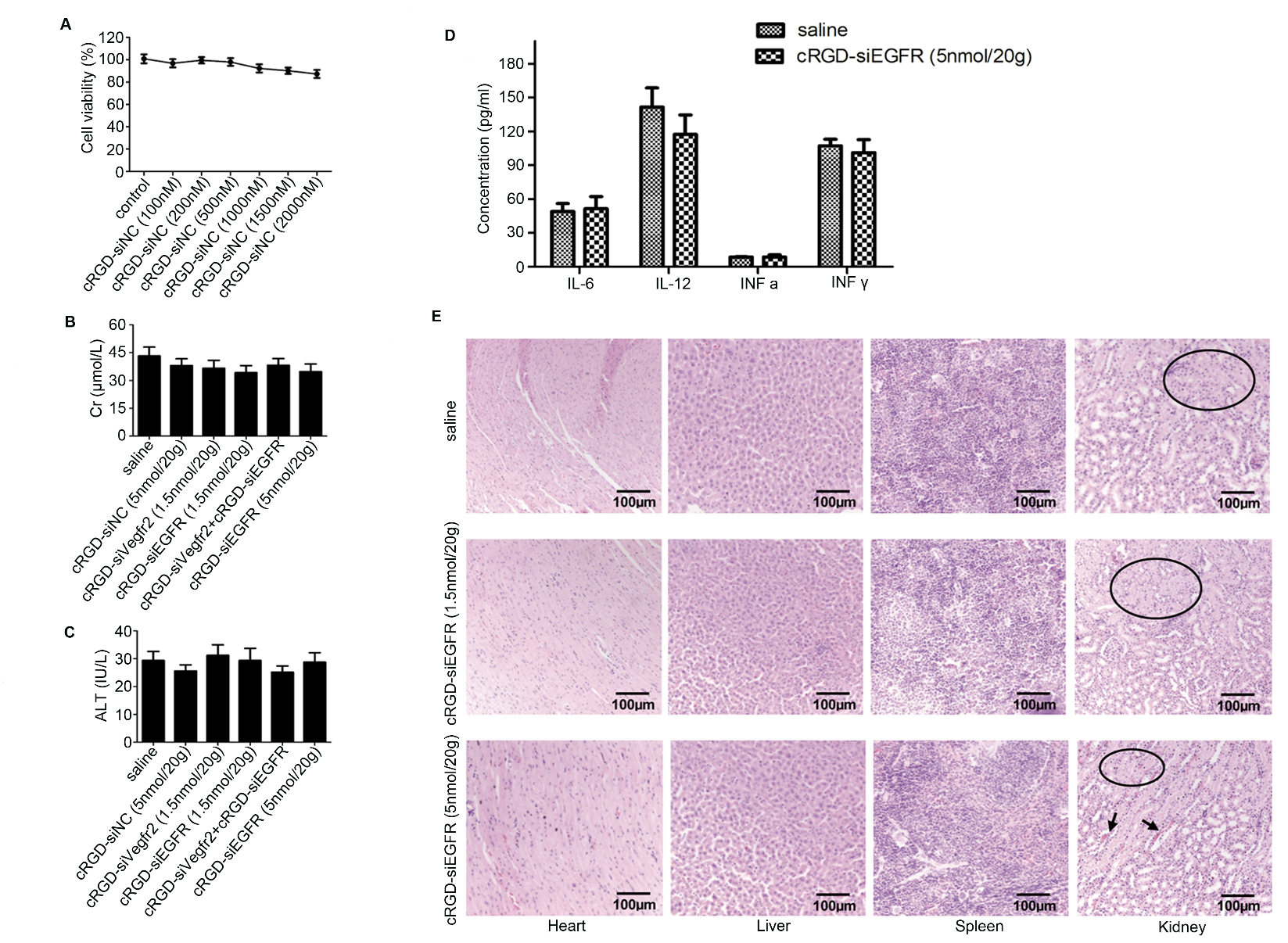


**Figure S2.** Toxicity and immunogenicity of cRGD-siEGFR. **(A)** cRGD-siRNA cytotoxicity in vitro. U87MG cells were incubated with cRGD-siNC at different concentration (100, 200, 500, 1000, 1500, or 2000 nM) for 24 h, and cell viability was analyzed using a CCK-8 assay. The Cr **(B)** and ALT **(C)** levels in serum. The nude mice were repeatedly injected with cRGD-siEGFR (7 times over a 48 h interval). Serum samples were prepared from mice 3 days after the last injection and measured by an automated Aeroset Chemistry Analyzer (Abbott, USA), based on the manufacturer’s instructions. **(D)** Analysis of IL-6, IL-12, IFN-α and IFN-γ levels in the serum of C57BL/6J mice 6 h after injection of cRGD-siEGFR (5 nmol/20 g) by ELISA. No differences were found between the groups. **(E)** The pathomorphological changes in nude mouse viscera. The samples were prepared from mice 3 days after the last injection and observed by HE staining. Hydropic degeneration of renal tubular epithelial cells was found in all three groups (indicated by roundness), and some renal tubules containing a small number of erythrocytes were observed only in the cRGD-siEGFR group (5 nmol/20 g) (indicated by the arrow); bar=100 µm.
